# Repassivation of Titanium Surface with Different Disinfectants After Implantoplasty: Effects on Corrosion, Ion Release, Cytotoxicity and Bacterial Behaviour—An In Vitro Study

**DOI:** 10.3390/dj14090587

**Published:** 2026-09-11

**Authors:** Leyre Millas, Javi Vilarrasa, Neus Carrió, Jose Nart, Javier Gil

**Affiliations:** 1 Department of Periodontology, Universitat Internacional de Catalunya, Carrer de Josep Trueta, s/n, 08195 Sant Cugat del Vallès, Spain; 2 Bioinspired Oral Biomaterials and Interfaces, Department of Science and Materials Engineering, Escola Enginyeria Barcelona Est., Universitat Politècnica de Catalunya, 08019 Barcelona, Spain; javier.gil.mur@upc.edu

**Keywords:** titanium, biofilm, peri-implantitis, surgical treatment

## Abstract

**Objective**: Implantoplasty smoothens exposed implant surfaces but disrupts the titanium oxide layer, reducing corrosion resistance, increasing ion release, and facilitating bacterial colonization. Restoring a stable and biologically favorable surface is therefore critical. Chemical agents capable of repassivating titanium may enhance surface integrity and antibacterial performance, yet the optimal agent remains unclear. This study evaluated the repassivation effects of common disinfectants on corrosion resistance, ion release, cytotoxicity, and antibacterial activity. **Methods**: One hundred twenty-eight discs underwent manual implantoplasty and were treated with citric acid (CA), ethylenediaminetetraace acid (EDTA), hydrogen peroxide (H_2_O_2_) or saline (S). Surface topography and wettability were analyzed using white-light interferometry and contact angle measurements. Corrosion behavior was assessed via open-circuit potential monitoring and potentiodynamic polarization in Hank’s solution. Titanium ion release was quantified over 21 days using inductively coupled plasma mass spectrometry. Cytotoxicity was evaluated in human fibroblast and osteoblast cultures exposed to sample extracts. Bacterial adhesion and viability of *Streptococcus sanguinis* and *Pseudomonas aeruginosa* were analyzed by scanning electron microscopy and confocal laser scanning microscopy after 24 h and 48 h. **Results**: H_2_O_2_ increased surface roughness, while CA and EDTA showed no effect. All agents enhanced hydrophilicity, with CA and H_2_O_2_ producing the greatest increase in surface energy. CA showed the highest corrosion resistance and lowest ion release. No treatment exhibited cytotoxicity, and all reduced bacterial adhesion, with CA and H_2_O_2_ showing superior bactericidal activity. **Conclusions**: CA and H_2_O_2_ improved surface properties after implantoplasty, with CA providing the most favorable combination of corrosion resistance, minimal ion release and antibacterial effect.

## 1. Introduction

Peri-implantitis is an inflammatory disease of bacterial origin affecting the tissues surrounding dental implants. It is characterized by inflammation of the peri-implant mucosa and the subsequent progressive loss of supporting bone [1], which, in the absence of treatment, appears to progress in a non-linear and accelerating pattern [2]. Furthermore, it is a complex and heterogeneous infection, associated not only with opportunistic bacterial pathogens but also with fungal organisms and viruses [3].

Decontaminating the biofilm adhered to the exposed implant surface is a primary therapeutic objective, since biofilm accumulation and bacterial dysbiosis represent the main etiological factors in peri-implantitis [4]. Achieving this goal remains one of the greatest clinical challenges; it requires both the effective disruption of the biofilm on the contaminated implant surface and the identification and management of local predisposing and precipitating factors involved in disease onset and progression.

In the pursuit of an effective treatment protocol to remove bacterial biofilms and resolve peri-implantitis, the literature has proposed a range of mechanical methods, chemotherapeutic agents, and combinations of both, evaluated through both in vivo and in vitro studies [5,6,7,8,9]. The optimal mechanical decontamination protocol should effectively remove hard deposits and bacterial plaque biofilms while avoiding any significant deleterious changes to the implant surface [10].

Implantoplasty—a procedure that mechanically removes the implant threads and smooths the modified surface—appears to be an effective adjunctive approach for the surgical treatment of peri-implantitis [11,12]. As widely reported in the literature, increased implant surface roughness favors biofilm accumulation [13,14,15], and rough surfaces promote bacterial adhesion and pathogenic biofilm formation. These biofilms can subsequently alter the electrochemical properties of the implant surface, increasing the likelihood of corrosion, while the resulting surface degradation further enhances bacterial colonization [16]. However, due to its subtractive nature, implantoplasty is associated with mechanical and biological complications both during the intervention and at later stages; these include the release of titanium particles into the surrounding tissues and a decrease in the implant’s corrosion resistance [17,18,19,20].

In this context, titanium passivation has been employed as a post-manufacturing process to clean the surface and promote the formation of a protective titanium oxide TiO_2_ layer, thereby enhancing corrosion resistance, reducing ion release, and generating a biologically favorable surface. Nonetheless, the impact of passivation and oxidative treatments—as well as the exact role of titanium oxide in modulating surface physicochemical properties—remains insufficiently characterized. Numerous approaches, including chemical agents, electrochemical methods, and laser-based protocols, have been investigated.

Traditional oral antiseptics, such as chlorhexidine or povidone-iodine, exert potent antimicrobial effects; however, they lack the chemical or oxidative capacity required to react with the underlying metallic lattice and restore the damaged titanium oxide (TiO_2_) passive layer. Conversely, agents possessing oxidative (H_2_O_2_) or acidic/chelating properties (citric acid, EDTA) can actively interact with the titanium surface, potentially driving chemical repassivation while simultaneously exerting antibacterial activity [21,22]. However, the comparative effectiveness of these specific repassivating agents after implantoplasty remains unclear. In addition, no consensus has been reached regarding an optimal chemotherapeutic agent capable of effectively balancing surface decontamination, enhanced corrosion resistance, minimal ion release, and reduced bacterial adhesion.

Because the adverse physicochemical consequences of implantoplasty may jeopardize the long-term stability of surgically treated implants—and no standardized protocol currently exists to reestablish the protective titanium oxide layer following surface modification—it is essential to determine whether chemical repassivation agents can successfully restore a stable, clinically favorable surface. Establishing this relationship is critical to ensure that the therapeutic benefits of implantoplasty are not counteracted by surface deterioration, which could otherwise predispose the implant to corrosive degradation, ion release, impaired tissue compatibility, and bacterial recolonization.

Therefore, the aim of this in vitro study was to analyze the repassivation effect of citric acid, EDTA, and hydrogen peroxide after implantoplasty on: (i) corrosion resistance, (ii) ion release, (iii) cytotoxicity, and (iv) bactericidal behavior.

## 2. Materials and Methods

### 2.1. Study Groups

A total of 568 commercially pure grade 3 titanium flat discs were included in the study. The discs (chemical composition: Ti 99.5%, O 0.3%, Fe 0.1%, C 0.05%, N 0.05% in weight percentage) were supplied by SOADCO SL (Klockner Medical Group, Escaldes-Engordany, Andorra) and measured 10 mm in diameter and 5 mm in thickness. Prior to experimental allocation, all specimens underwent standardized surface conditioning by sandblasting with 45 µm aluminum oxide (Al_2_O_3_) particles, applied at a pressure of 2.5 bar and a fixed nozzle-to-sample distance of 70 mm. The selection of these specific chemical agents was based on their combined potential for chemical repassivation of the titanium oxide (TiO_2_) layer and surface decontamination, as opposed to conventional non-reactive antiseptics (e.g., chlorhexidine or povidone-iodine), which lack oxide-regenerating chemical properties.

The discs were randomly distributed into four experimental groups according to the post-implantoplasty repassivation protocol:

1. Implantoplasty + saline (I + S) (*n* = 32), serving as the control group
2. Implantoplasty + 40% citric acid (I + CA) (*n* = 32)
3. Implantoplasty + EDTA (I + EDTA) (*n* = 32)
4. Implantoplasty + hydrogen peroxide (I + H_2_O_2_) (*n* = 32)

### 2.2. Sample Preparation

#### 2.2.1. Implantoplasty Procedure

Each titanium disc was fixed in a custom-made resin holder to ensure stability during instrumentation. Implantoplasty was performed manually by a single experienced operator (LM) under 2.8× magnification loupes with integrated LED illumination (Akura, Novamed Concepts SL, Madrid, Spain). The procedure was carried out using a force-controlled dynamometer to apply consistent pressure between the drill and the titanium surface. The applied force was maintained at a constant load of 10 N using an automatic dynamometric control system, corresponding to the mean force measured across 59 clinicians at the Universitat Internacional de Catalunya’s University Dental Clinic. The application of the load was 60 s for each disc. The surface of the samples was sequentially modified by using a GENTLEsilence LUX 8000B turbine (KaVo Dental GmbH, Biberach, Germany) with continuous irrigation, using either a coarse-grained diamond bur (ref: 863-010F-FG, NTI, Kahla, Germany) or a fine-grained WC-bur (ref: H48-L-014-FGXXL, NTI, Kahla, Germany) as milling tools, respectively. To ensure consistent cutting performance, burs were replaced after every 20 specimens.

#### 2.2.2. Repassivation Process

Immediately after implantoplasty, specimens were treated for 1 min with one of the following solutions: saline, citric acid, hydrogen peroxide, or EDTA, according to group allocation. Subsequently, all samples were subjected to a standardized ultrasonic cleaning protocol consisting of three consecutive 3 min cycles: two in distilled water and one in ethanol.

### 2.3. Characterization of the Sample After Treatment

#### 2.3.1. Surface Roughness

Surface topography was evaluated using a white-light interferometer (Wyko NT1100, Veeco Instruments, Plainview, NY, USA) operating in vertical scanning interferometry mode, which provides a vertical resolution of approximately 2 µm. Measurements were obtained over a scanned area of 124.4 × 94.6 µm for six samples for each treatment. Data processing was performed using Wyko Vision 32 software (Veeco Instruments, USA), applying a Gaussian filter to isolate surface roughness from waviness and form. Five specimens per experimental condition were analyzed, and the arithmetic mean height (Sa) and the maximum height (Sz) were recorded.

#### 2.3.2. Contact Angle and Surface Free Energy

Static contact angle measurements were performed using ultrapure distilled water (Millipore Milli-Q, Merck Millipore Corporation, Darmstadt, Germany) and formamide on six specimens per group with a contact angle analyzer (OCA15plus, Dataphysics, Filderstadt, Germany). Droplets of 3 μL (water) and 1 μL (formamide) were dispensed at a flow rate of 200 μL/min using a micrometric syringe. Each measurement was repeated three times per specimen, and data acquisition and analysis were performed with SCA20 software (Dataphysics, Filderstadt, Germany). Surface free energy (SFE) was calculated using the Owens–Wendt–Rabel–Kaelble (OWRK) method according to the following equation [23]:

γL1+cosθ=2γLdγSd12+γLpγSp12

where *γ_S_* is the surface tension of the solid (S), *γ_L_* the surface tension of the liquid (*L*), *γ_SL_* the interfacial free energy or SE between *L* and *S*, *θ* the contact angle between *L* and *S*, and *γ^d^* and *γ^p^* represent the dispersive and polar components of the SE, respectively. Where is due to dipole–dipole interactions (London or ‘dispersion’), and is the polar component produced by the permanent interaction between dipoles.

### 2.4. Corrosion Tests

Corrosion behavior was evaluated on ten specimens per group, each providing an exposed surface area of 19.6 mm^2^. Hank’s balanced salt solution (ThermoFisher, Madrid, Spain) was used as the electrolyte due to its physiological ionic composition, as previously described [24]. Electrochemical testing was performed in a sterile 200 mL polypropylene cell fitted with a polymethyl methacrylate lid accommodating six electrode ports. A saturated calomel electrode (SCE; +0.241 V vs. SHE) was used as the reference electrode, platinum served as the counter electrode, and the titanium discs served as the working electrode. All measurements were conducted at room temperature within a Faraday cage to minimize electromagnetic interference.

Open-circuit potential (OCP) was monitored for 5 h using a two-electrode configuration, with potential values recorded every 10 s. Stabilization was defined according to ASTM G31 criteria as a potential variation of less than 2 mV over a 30 min interval [25]. Higher OCP values were interpreted as indicative of greater corrosion resistance. Data acquisition and E–t curve analysis were performed using PowerSuite software (version 3.0).

Following OCP stabilization, cyclic potentiodynamic polarization tests were carried out using a three-electrode setup in accordance with ASTM G5 standards [26]. Polarization scans ranged from −0.8 V to +1.7 V relative to the stabilized EOCP at a scan rate of 2 mV/s. Electrochemical parameters, including corrosion potential (Ecorr, mV), corrosion current (Icorr, μA), current density (jcorr, μA/cm^2^), and polarization resistance (Rp, Ω), were extracted using the PowerCorr–Cyclic Polarization module. Tafel slopes were calculated following ASTM G102 guidelines [27]. Polarization resistance values were subsequently used to estimate corrosion rates via the Stern–Geary equation, assuming a constant K value of 0.025.

### 2.5. Ion Release Tests

Ion release into Hank’s solution was quantified in accordance with ISO 10993-12 guidelines [28], using a ratio of 1 mL of extraction medium per 0.2 g of material. Each specimen was immersed in 5 mL of solution and incubated at 37 °C for 1, 3, 7, 14, and 21 days. Ten samples for each time were studied. At each time point, 2.5 mL of the medium was collected and replaced with fresh solution, applying dilution corrections. Collected extracts were filtered through 0.22 μm membranes, acidified with 2% ultrapure nitric acid, and analyzed by inductively coupled plasma mass spectrometry (ICP-MS, PerkinElmer Elan 6000, Shelton, CT, USA), with detection limits in the parts-per-trillion range. Results were expressed as parts per billion (ppb).

### 2.6. Cell Viability

Indirect cytotoxicity testing was performed in accordance with ISO 10993-5 [29]. Ten specimens were incubated at 37 ± 2 °C for 72 h and for each treatment to obtain extraction media at concentrations of 100%, 50%, 10%, 1%, and 0.1%. Human foreskin fibroblasts (HFFs) (ThermoFisher C0135C Waltham, MA, USA) were seeded at a density of 5000 cells per well in 96-well plates. After 24 h, the culture medium was replaced with the corresponding extracts and incubated for an additional 24 h. Cell lysis was induced by adding 200 µL of mammalian protein extraction reagent (M-PER^®^) per well. Lactate dehydrogenase (LDH) release was quantified using a commercial cytotoxicity detection kit (ThermoScientific, Waltham, MA, USA), and absorbance was measured at 490 nm with a microplate reader (ELx800, Bio-Tek Instruments, Charlotte, VT, USA). Cytotoxicity percentages were calculated using standard reference controls. All assays were performed in triplicate.

The objective of osteoblast cultures is to determine the degree of osteoblastic cytocompatibility and the ease of hard tissue regeneration to achieve bone regeneration and increase the mechanical fixation of the implant–abutment system to the bone with osseointegration. For the cell adhesion assay, osteoblastic SaOS-2 cells (Merck code 41106514, Darmstadt, Germany), a cell line with epithelial morphology derived from bone, were used. Six to seven cell passages were performed before seeding the cells onto the study samples. During cell passages, a control of the growth and cell viability was tested. Cells were initially thawed by gently shaking the cryovial in a 37 °C water bath for 1–2 min. From this point onward, everything was performed under sterile conditions. Once thawed, the content was transferred to a falcon with 9 mL of culture medium and centrifuged at 300 G for 3 min. Then, the supernatant was aspirated, and the pellet was resuspended with 1 mL of cell culture medium. Cells were seeded in flasks F175 and were kept at 37 °C with 5% CO_2_, with the cell culture medium being changed 2–3 times per week.

The cell culture medium for this cell line consists of McCoy’s 5a Medium Modified with L-glutamine 1.5 mM and 2200 mg/L sodium bicarbonate. This medium was supplemented with 15% fetal bovine serum (FBS), 1% penicillin/streptomycin (P/S) and 2% sodium pyruvate solution (NaPyr).

Cell passage was carried out at 90% confluence. Therefore, the cells were detached from the flasks by removing the medium, washing twice with 5 mL of PBS (37 °C) to remove dead cells, and then adding 5 mL of 0.05% trypsin. The flasks were left in the incubator at 37 °C with 5% CO_2_ for 2–3 min. Afterward, trypsin was neutralized with 7 mL of cell culture medium (37 °C), and the content was transferred to a Falcon tube and centrifuged for 5 min at 300 G. The supernatant was then aspirated, and the pellet was resuspended with cell culture medium. Cell counting with Tripan Blue was then performed. For this, 10 μL of previously resuspended cells was prepared in an Eppendorf tube and mixed with Tripan Blue. A volume of 10 μL of the total was transferred to a Neubauer chamber for counting using phase contrast microscopy. The corresponding calculations were then carried out [30,31]. Ten samples for each treatment were studied.

### 2.7. Bacterial Cultures

#### 2.7.1. Bacterial Strains

Ten specimens from each experimental group were used for microbiological analysis. Two reference strains were selected: *Streptococcus sanguinis* (CCUG 59325) (Culture Collection University Gothenburg, Gothenburg, Sweden) as a representative Gram-positive early colonizer, and *Pseudomonas aeruginosa* (CCUG-EUCPA) (Culture Collection University Gothenburg, Gothenburg, Sweden) as a representative Gram-negative bacterial strain, both relevant to early peri-implant biofilm formation.

#### 2.7.2. Bacterial Growth and Inoculation

Bacterial cultures were obtained by incubating agar plates at 37 °C for 24 h. Colonies were then suspended in Brain Heart Infusion broth and incubated for an additional 24 h. The bacterial suspension was adjusted to an optical density of OD600 = 0.1. For adhesion assays, 500 µL of the standardized suspension was applied to each specimen and incubated under static conditions at 37 °C for one hour. After incubation, samples were gently rinsed with phosphate-buffered saline (PBS), fixed in 2.5% glutaraldehyde at 4 °C for 30 min, and washed again with PBS.

#### 2.7.3. Adhesion and Viability Analysis

For scanning electron microscopy (SEM), specimens were dehydrated through a graded ethanol series, air-dried, and sputter-coated with Pt-Pd layer. Ten micrographs per sample were obtained at 20,000× magnification, and bacterial adhesion was quantified as colony-forming units (CFUs) per specimen.

Bacterial viability was assessed using the LIVE/DEAD™ BacLight™ Bacterial viability kit (Thermo Fisher, Spain). Samples were stained with propidium iodide, incubated in the dark, rinsed with PBS, and analyzed by confocal laser scanning microscopy at 63× magnification using excitation wavelengths of 488 nm and 561 nm.

### 2.8. Statistical Analysis and Number of Samples

Descriptive statistics were reported as means and standard deviations (SD). Data distribution normality and homogeneity of variances were assessed using the Shapiro–Wilk and Levene tests, respectively. For intergroup comparisons, analysis of variance (ANOVA) followed by Tukey’s multiple comparison test was used to assess statistically significant differences between groups. Statistical analysis was performed using SPSS software (version 20.0; SPSS Inc., Chicago, IL, USA). Statistical significance was set at *p* < 0.05.

To determine the sample size, the following method was used: ‘Inference for Means: Comparing Two Independent Samples’ website developed by the Department of Statistics at the Univ. of British Columbia (http://www.stat.ubc.ca/~rollin/stats/ssize/n2.html accessed 10 April 2026) [32]. A sample size of 6 was calculated for a desired power of 0.94 and significance of *p*-value < 0.05 was. In this work, we indeed tested 10 samples, which is a size larger than the minimum necessary for the power desired for the cellular and microbiologic studies. For the roughness and wettability studies we used 6 samples for each treatment but different tests were realized in each sample.

Roughness: 6 samples × 4 treatments = 24 samples

Wettability. 6 samples × 4 treatments = 24 samples

Corrosion. 10 samples × 4 treatments × 2 (Open and potentiodynamic) = 80 samples

Ion release: 10 samples × 4 treatments × 5 times = 200 samples.

Cellular viability: 10 samples × 4 treatments × 2 (HFFs and SaOS-2) = 80 samples

Microbiological studies: 10 samples × 4 treatments × 2 bacteria strains × 2 tests = 160 samples.

TOTAL: 568 samples

## 3. Results

### 3.1. Characterization of the Surface

#### 3.1.1. Surface Roughness

**Figure 1:**
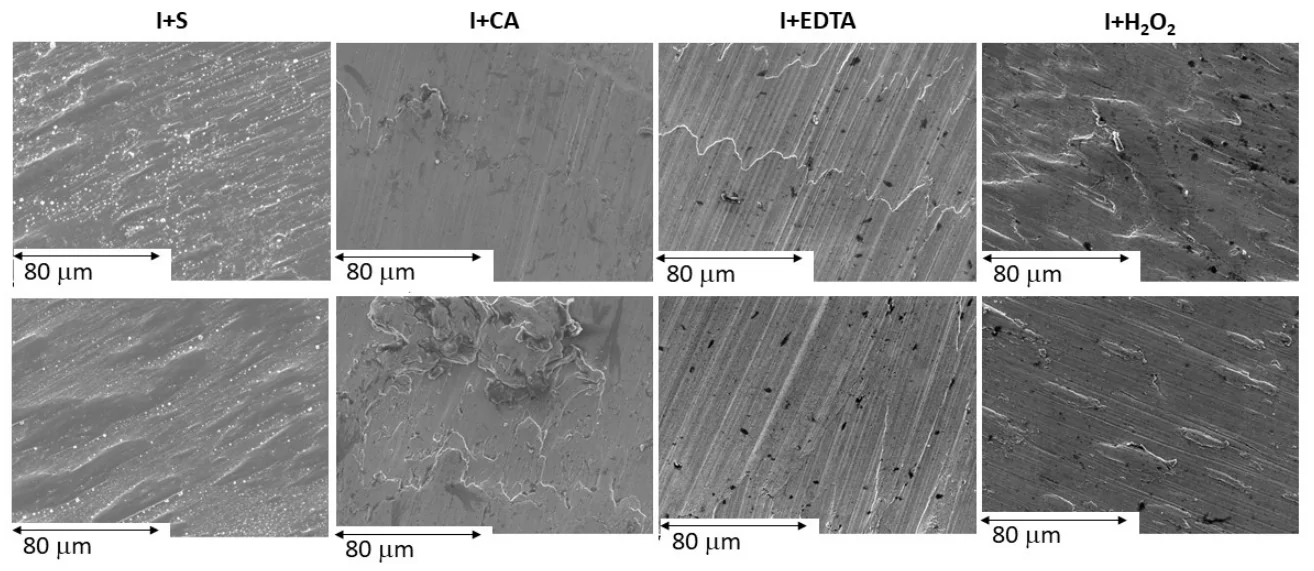
Depiction of the surface morphology of titanium discs observed by electron microscopy following the passivation process.

SEM images illustrate the surface topography of the titanium discs after the passivation process (Figure 1). No significant differences in surface characteristics were observed among the discs treated with the various passivation solutions.

The roughness analysis revealed that only the I + H_2_O_2_ treatment significantly increased the surface roughness of titanium compared to the control (I + S) (*p* < 0.05). In contrast, the I + CA and I + EDTA treatments did not induce significant changes, showing roughness values comparable to or lower than those of the control group (Table 1).

#### 3.1.2. Contact Angle and Surface Free Energy

**Figure 2:**
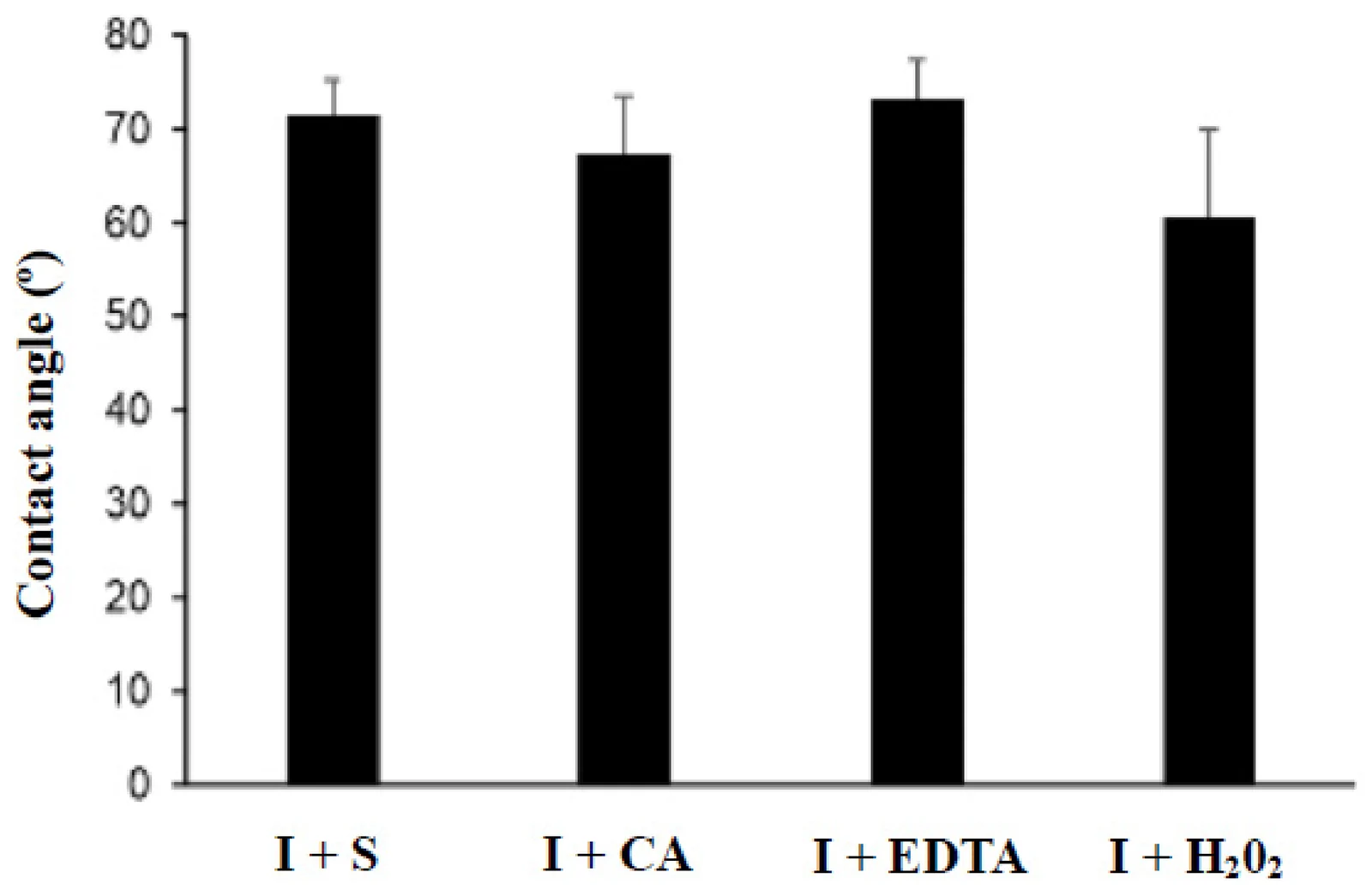
Contact angle values.

All evaluated treatments exhibited a hydrophilic surface, as evidenced by contact angle values below 90° (Figure 2). However, no significant differences in wettability were detected among the surface treatments (*p* > 0.05).

Table 2 summarizes the SFE values for each group. The control group (I + S) showed a low polar component and a predominantly dispersive surface profile. In contrast, the I + CA and I + H_2_O_2_ treatments significantly increased both the polar component and total SFE compared with the control (*p* < 0.05). The I + EDTA treatment showed no significant differences, with values similar to those of the control.

### 3.2. Corrosion Behavior

The electrochemical parameters derived from the potentiodynamic polarization curves and corresponding Tafel slopes are summarized in Table 3. The I + CA group exhibited superior corrosion resistance, evidenced by a lower Icorr, reduced corrosion rate, and higher Rp. Although the I + H_2_O_2_ group displayed a comparable corrosion rate, its lower Rp indicates greater susceptibility to minor potential fluctuations.

**Figure 3:**
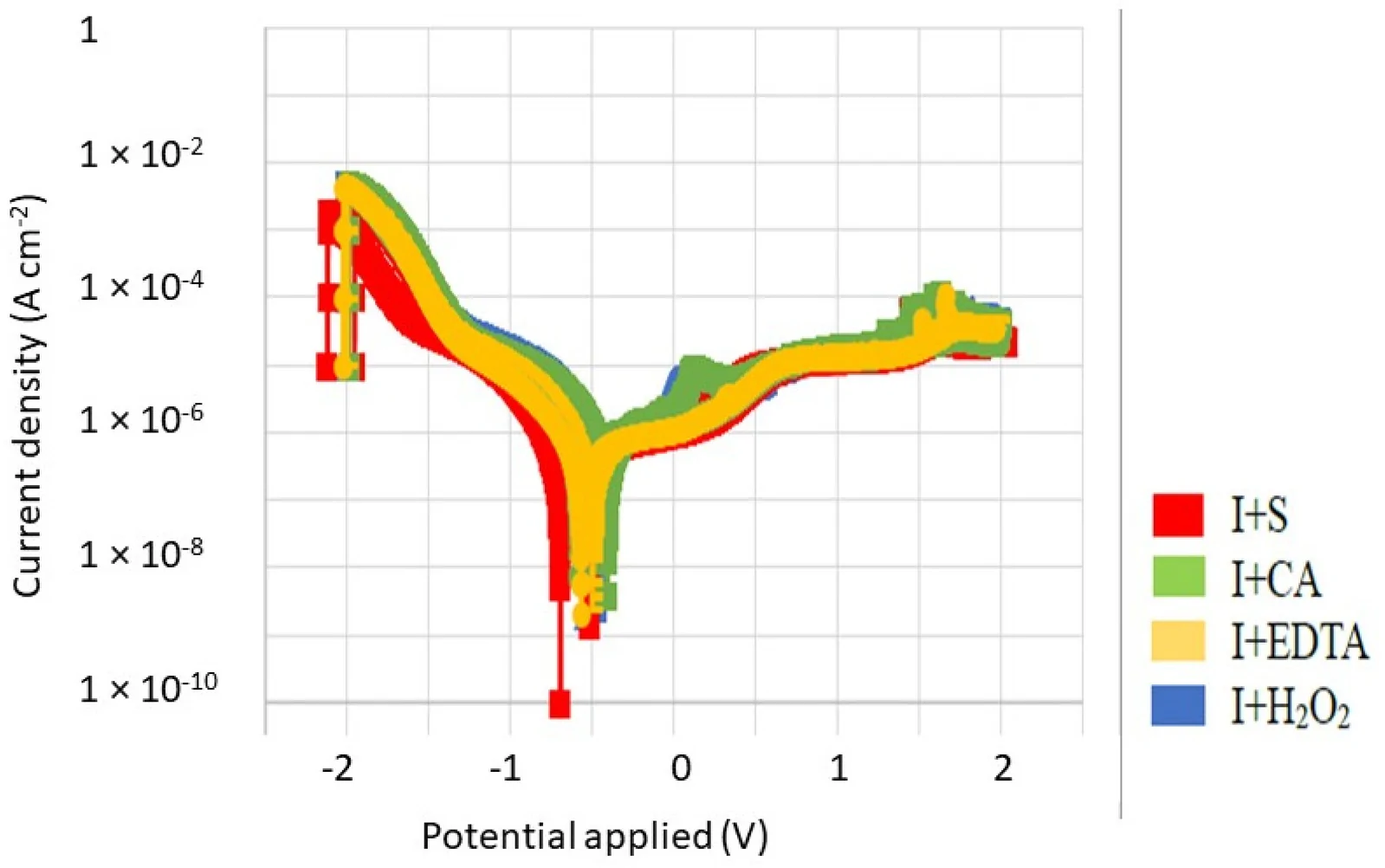
Potentiodynamic polarization curves of titanium surfaces after different repassivation treatments.

As illustrated in Figure 3, both I + CA and I + H_2_O_2_ treatments resulted in a lower corrosion intensity, corresponding to a decreased corrosion rate. The oxidative nature of these agents promotes further oxidation of the titanium surface, forming a thicker and more stable oxide layer. This enhanced passive film improves the overall corrosion resistance of the material.

### 3.3. Ion Release

Table 4 presents the ion release values from titanium discs after 21 days of immersion in Hank’s solution for each repassivation treatment. The I + CA group consistently exhibited the lowest ion release, with statistically significant differences compared to the control (I + S) at all time points (*p* < 0.05). The I + EDTA group showed ion release levels similar to the control, except at day 14, where a significant increase was observed relative to both the control and I + CA groups (*p* < 0.05). In contrast, the I + H_2_O_2_ group demonstrated the highest ion release from day 7 onward, reaching a peak at day 14 that was significantly higher than in all other groups (*p* < 0.05).

### 3.4. Cell Viability

The cytotoxicity results are presented in Table 5 for fibrobalsts and Table 6 for osteblasts. No statistically significant differences were detected among groups at any dilution (*p* > 0.05). All treatments maintained high cell viability across all concentrations, indicating the absence of cytotoxic effects on HFF cells and SaOs-2 under the tested conditions.

### 3.5. Bacterial Adhesion

The CFU counts of *P. aeruginosa* and *S. sanguinis* after 24 and 48 h are summarized in Table 7. All experimental treatments (I + EDTA, I + CA and I + H_2_O_2_) produced a significant reduction in bacterial viability compared to the control (I + S) at both time points (*p* < 0.05).

For *P. aeruginosa*, CFU values were significantly lower in all treatment groups relative to the control at both 24 and 48 h, with the I + CA group exhibiting the lowest bacterial counts at 48 h (0.98 ± 0.07).

For *S. sanguinis*, the I + CA and I + H_2_O_2_ treatments yielded the most pronounced reductions, with CFU values significantly lower than those of the control and the I + EDTA groups at both 24 and 48 h. At 48 h, the I + H_2_O_2_ treatment demonstrated the lowest CFU count, showing statistically significant differences compared with all other groups.

### 3.6. Bacterial Viability

**Figure 4:**
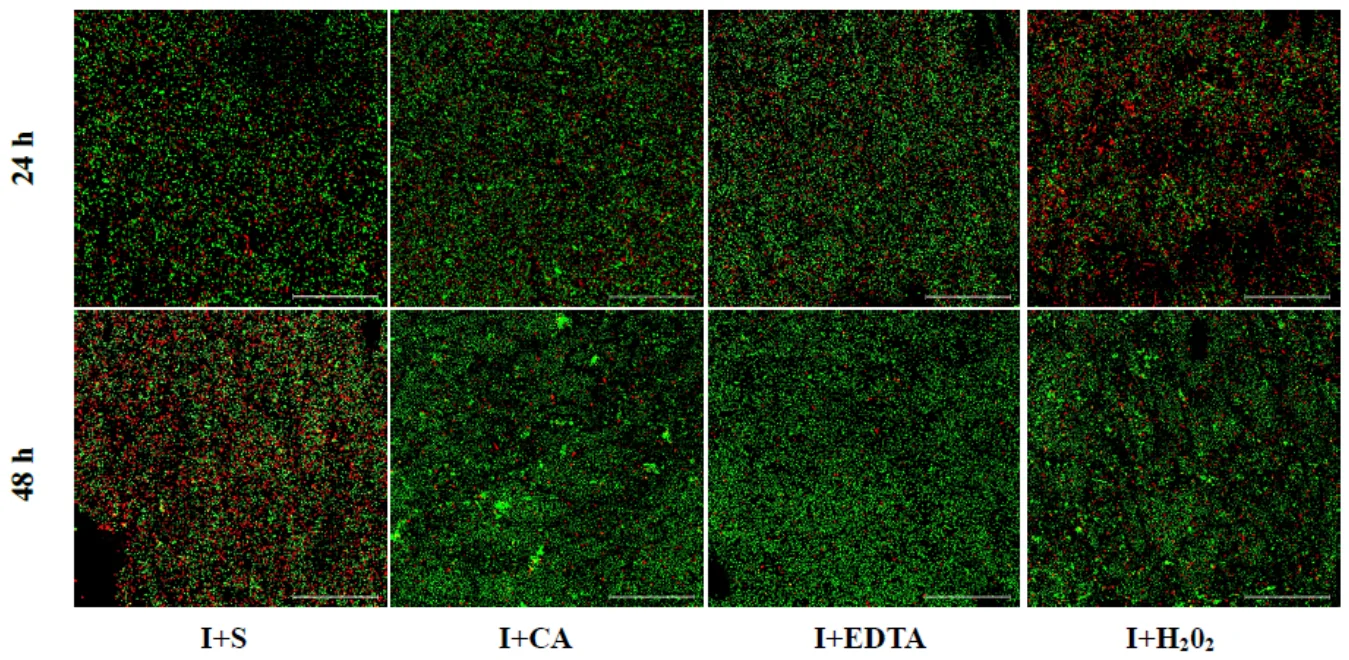
CLSM images of *P. aeruginosa* stained with the LIVE/DEAD™ BacLight™ Bacterial viability kit. Live bacteria are marked in green, and dead bacteria are marked in red.

**Figure 5:**
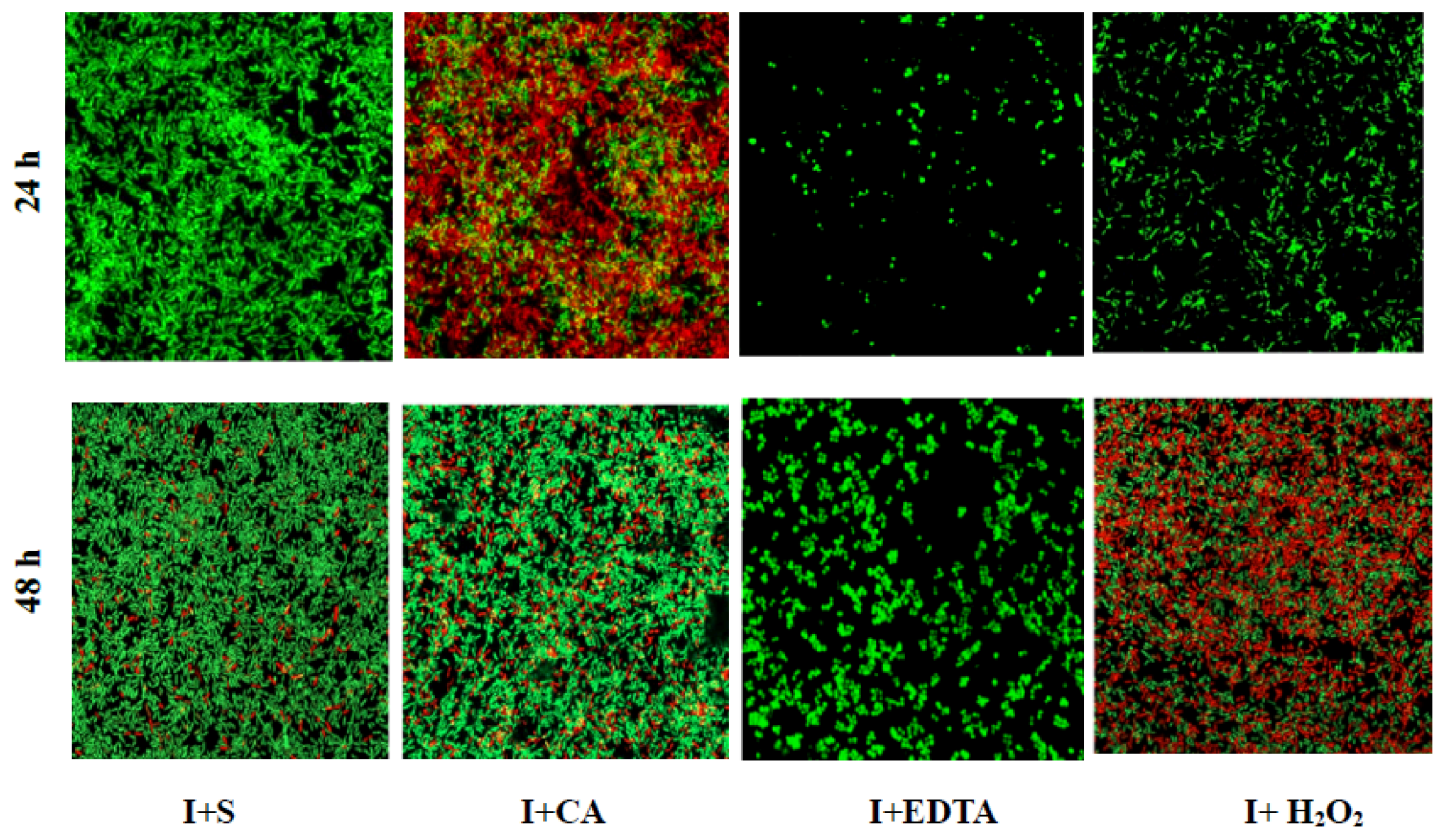
CLSM images of *S. sanguinis* stained with LIVE/DEAD™ BacLight™ Bacterial viability kit. Live bacteria are marked in green, and dead bacteria are marked in red.

CLSM analysis revealed marked differences in bacterial viability among the treatment groups. Surfaces treated with I + CA and I + H_2_O_2_ exhibited a pronounced increase in red-stained *P. aeruginosa* cells at 24 and 48 h, respectively, indicating enhanced bactericidal activity. In contrast, the I + S and I + EDTA treatments showed minimal antibacterial effects, suggesting limited efficacy in preventing *P. aeruginosa* adhesion (Figure 4). Quantitative analysis (Table 8) corroborated these observations, demonstrating a significantly higher proportion of dead bacteria in the I + CA and I + H_2_O_2_ groups. For *S. sanguinis*, the strongest bactericidal effects were observed in the I + CA group at 24 h and in the I + H_2_O_2_ group at 48 h. Conversely, I + S and I + EDTA surfaces exhibited limited activity against this strain (Figure 5). Quantitative analysis (Table 7) further supported these findings, showing a greater proportion of dead bacteria on surfaces treated with I + CA and I + H_2_O_2_ compared with the other groups.

## 4. Discussion

Implantoplasty is widely performed to reduce implant surface roughness, limit bacterial colonization, and improve clinical outcomes [33,34,35,36]. However, by mechanically removing the implant’s original microtopography, the procedure also exposes fresh titanium susceptible to corrosion, ion dissolution, and altered surface energy. Previous in vitro studies have shown that implantoplasty modifies electrochemical behavior [17,37], and several studies [6,38] have examined how chemical agents influence bacterial adhesion and surface stability. Yet, despite their frequent empirical use, the combined effects of mechanical polishing and subsequent chemical repassivation remain poorly characterized. The present study demonstrates that not all chemical agents behave similarly after implantoplasty and that their impact on corrosion resistance, ion release, and antibacterial performance varies markedly.

The solutions were applied for 1 min, consistent with exposure times previously employed in implant surface decontamination studies [11,39]. Among the tested agents, citric acid (CA) provided the most balanced and favorable response. Its application enhanced hydrophilicity, minimized 21-day titanium ion release (8.0 ppb), increased corrosion resistance, and exerted strong bactericidal effects—particularly against *S. sanguinis*—without increasing surface roughness or compromising cytocompatibility. These findings reinforce a growing body of literature supporting the beneficial effects of CA on titanium surfaces [6,24,40,41,42]. Although the exact mechanism through which CA enhances surface reactivity remains unclear, its ability to modulate surface energy, promote clot adhesion, interact with bacterial membranes, and generate a more stable passive oxide layer likely contributes to the improved electrochemical and antibacterial profiles observed [11].

Hydrogen peroxide H_2_O_2_ also demonstrated a robust antibacterial effect, aligning with its known mechanism of generating reactive oxygen species and inducing oxidative cell damage [43]. However, its overall performance was less consistent due to a significant increase in surface roughness. While this did not translate into higher bacterial counts within the evaluation period, 48 h may be insufficient to fully assess the influence of surface roughness on mature biofilm accumulation. Early bacterial adhesion is strongly driven by surface chemistry and physicochemical interactions, whereas the effect of roughness becomes more pronounced during later stages of biofilm maturation, where surface irregularities provide niches for bacterial retention and growth. Furthermore, the increased roughness observed with H_2_O_2_ was associated with substantially higher ion release (23.8 ppb), indicating potential micro-etching and reduced stability of the protective oxide layer. These findings highlight a critical distinction: strong antimicrobial activity does not necessarily ensure a beneficial or stable repassivated surface.

Conversely, EDTA provided moderate antibacterial effects and showed limited impact on corrosion resistance or surface energy. As a chelating agent, EDTA is known to disrupt biofilms [44,45], but its performance here suggests that its interaction with freshly machined titanium is insufficient to confer meaningful physicochemical advantages. Saline, used as a control, showed the lowest antibacterial capacity and offered no protective improvements to the titanium surface.

The electrochemical findings are equally revealing. Implantoplasty alone produces machining-induced defects, such as microgrooves and microcracks, which serve as initiation sites for corrosion [17,37]. This is consistent with the higher corrosion intensity and lower polarization resistance observed in our control group. Chemical repassivation clearly mitigated these defects; CA produced the most stable passive layer, as evidenced by its low corrosion current density and high polarization resistance. These results align with a previous study that reported improved corrosion resistance in implantoplasty-treated titanium following CA immersion [46,47].

The biological and mechanical implications of implantoplasty extend beyond immediate surface decontamination. The mechanical subtraction of titanium threads inevitably releases metallic debris into the peri-implant environment. Particle size plays a pivotal role in biological reactivity; while micro-particles may be encapsulated by fibrous tissue, nanometric titanium particles pose a higher toxicological concern, as they can evade physiological clearance mechanisms, become phagocytosed by macrophages, and trigger a pro-inflammatory cascade that accelerates peri-implant bone loss [17,19]. Additionally, the reduction in structural wall thickness following thread removal compromises the mechanical behavior and fatigue strength of the implant body [19,20].

Regarding titanium ion release, our findings indicate that 40% citric acid treatment (I + CA) resulted in the lowest ion dissolution over a 21-day period (8.0 ppb), significantly outperforming the control and hydrogen peroxide groups. While some previous reports suggest that citric acid may increase titanium release through acidic surface etching, such outcomes are primarily associated with prolonged exposure times or unrinsed acidic residues [48]. In contrast, our protocol employed a short 1 min application followed by thorough ultrasonic cleaning in ethanol and distilled water. This controlled chemical exposure removes unstable oxides created by mechanical implantoplasty and promotes the rapid formation of a uniform, corrosion-resistant TiO_2_ passive layer, thereby suppressing long-term ion leaching. Importantly, the clinical relevance of these measurements must be interpreted in light of their absolute magnitude. The ion release observed across all groups ranged strictly within parts per billion (ppb), approaching the lower detection limits of ICP-MS analytical sensitivity. In contrast to other dental biomaterials—such as orthodontic NiTi or beta-titanium archwires, which typically exhibit ion release in the parts per million (ppm) range [49]—the concentrations detected here are physiologically negligible. This explains the absence of cytotoxic effects observed in our HFF and SaOS-2 cell cultures and confirms that repassivation with citric acid effectively stabilizes the titanium surface without compromising biological safety.

The lack of correlation between reduced corrosion resistance and ion release may be explained by the distinct mechanisms underlying each phenomenon. Corrosion resistance relates primarily to the stability of the protective titanium oxide layer, whereas ion release is associated with the liberation of titanium ions into the physiological environment following bond disruption. Interestingly, previous studies on non-implantoplasty surfaces [24,40] reported that CA produced the highest ion release, likely due to acid-induced roughening. These divergent outcomes demonstrate that the effects of chemical agents cannot be dissociated from the prior mechanical state of the surface: the same agent may stabilize a polished surface while destabilizing a roughened one.

All treatments maintained high human foreskin fibroblast (HFF) and osteoblast viability, indicating that CA, H_2_O_2_, and EDTA are non-cytotoxic under the tested conditions. These findings align with previous evidence supporting the cytocompatibility of CA [6,39,50], H_2_O_2_ [51], and EDTA [6] with other cell lines, such as osteoblastic cells. However, reduced osteoblast viability has been observed previously with 30% CA [47], as well as with H_2_O_2_ and EDTA [50]. These discrepancies in the literature underscore that the biological response to these agents may be concentration-dependent, highlighting the need to define standardized concentrations and application protocols to ensure predictable biological outcomes.

All tested chemical agents significantly reduced bacterial viability compared to saline, underscoring their potential value as adjunctive decontamination strategies following implantoplasty. This aligns with previous findings showing that untreated titanium surfaces permit rapid colonization [11], a trend reflected in the elevated colony-forming unit (CFU) levels of our saline group. Pseudomonas aeruginosa and Streptococcus sanguinis were selected for this study because they are recognized as primary colonizers involved in the early stages of biofilm formation on titanium surfaces [24,40,41].

Among the agents, CA displayed the strongest early bactericidal effect, producing the greatest reductions in *P. aeruginosa* and *S. sanguinis* at 24 h. By 48 h, H_2_O_2_ emerged as the most effective compound—particularly against *S. sanguinis*—indicating that its antimicrobial action may be more sustained over time. These outcomes may be attributed not only to the intrinsic antimicrobial properties of acids and peroxides but also to the physicochemical alterations they induce on titanium surfaces. CA, with its low pH and chelating ability, disrupts bacterial membrane integrity and may destabilize surface-adhered proteins essential for early colonization [52,53]. This interpretation is consistent with multiple in vitro and in situ studies reporting robust antibacterial performance of CA in both single- and multi-species oral biofilms [41,42,43,54].

Nevertheless, clinical investigations have shown minimal differences in the microbiological profiles of CA-coated versus control abutments [55], suggesting that the strong in vitro efficacy of CA may be moderated in vivo by factors such as biofilm maturity, host response, and application feasibility. In contrast, H_2_O_2_ exerts oxidative damage that preferentially affects Gram-negative bacterial membranes [56], explaining the high proportion of dead *P. aeruginosa* detected. The comparatively weak antibacterial effect of EDTA is coherent with its limited acidity, lack of oxidative potential, and minimal influence on SFE.

To contextualize these findings within the current literature, our results must be compared with both clinical trials and existing in vitro evidence. Clinically, surgical implantoplasty has demonstrated favorable long-term outcomes in resolving peri-implant inflammation and arresting bone loss [35,57,58]. However, clinical studies predominantly focus on macroscopic clinical parameters—such as probing pocket depth, bleeding on probing, and radiographic bone levels—without assessing the electrochemical degradation, surface energy, or ionic dissolution of the modified titanium substrate [58,59]. On the other hand, conflicting in vitro reports exist regarding the chemical impact of repassivating agents. While some investigators report that citric acid treatment may cause surface etching and elevated titanium ion release, such detrimental effects are largely attributed to prolonged acidic exposure or unrinsed chemical residues that continuously solubilize the oxide film. Conversely, our short 1 min application followed by standardized ultrasonic cleaning aligns with studies demonstrating that controlled citric acid exposure effectively removes unstable milling oxides and promotes the formation of a homogeneous, corrosion-resistant TiO_2_ passive layer. Similarly, while hydrogen peroxide (H_2_O_2_) is widely praised in clinical literature for its potent antimicrobial action, conflicting laboratory findings show that strong oxidative attack can compromise surface smoothness and accelerate long-term ion release, a phenomenon confirmed by our topographical and ICP-MS results [60]. Reconciling these clinical and laboratory observations underscores that chemical repassivation after implantoplasty must strike a delicate balance between potent antimicrobial action and the preservation of the material’s electrochemical stability.

This study has limitations inherent to in vitro research. The simplified laboratory environment does not replicate the dynamic, multi-species conditions of the oral cavity. Additionally, the limited number of bacterial strains, non-probabilistic sample selection, and small sample sizes in certain assays may reduce the generalizability of the findings.

Nonetheless, the clear differences observed across treatments substantiate that chemical repassivation should not be considered a uniform process, but rather a critical factor determining the functional success of implantoplasty. Future research should incorporate analyses of both osteoblasts and epithelial cells to better characterize the cellular effects of implantoplasty treatments, utilize multi-species biofilm models, perform in vivo evaluations, and develop clinically practical CA formulations to further validate and refine these findings.

## 5. Conclusions

Within the limitations of this in vitro study, 40% citric acid (CA) provided the most favorable overall outcome for titanium repassivation following implantoplasty, effectively restoring corrosion resistance, minimizing titanium ion release, and exerting strong bactericidal activity without altering surface roughness or causing cytotoxicity. Although H_2_O_2_ displayed potent bactericidal properties, it adversely increased surface roughness and titanium ion release over time. Clinical in vivo trials are required to confirm these preliminary laboratory findings.

## Figures and Tables

**Table 1:** Roughness parameter values of the treated titanium samples.

	Sa (µm)	Sz (µm)
I + S	0.40 ± 0.09	0.75 ± 0.12
I + CA	0.32 ± 0.10	0.60 ± 0.19
I + EDTA	0.42 ± 0.11	0.48 ± 0.10
I + H_2_O_2_	0.55 ± 0.13	1.02 ± 0.18 *

* *p* value < 0.05 compared to control (I + S).

**Table 2:** Components of the surface free energy for implantoplasty-treated surfaces after different passivation treatments.

	*γ^d^* (mJ/m^2^)	*γ^p^* (mJ/m^2^)	SFE (mJ/m^2^)
I + S	35.15 ± 1.28	1.12 ± 0.10	36.27 ± 1.35
I + CA	30.08 ± 3.28 *	11.65 ± 3.28 *	41.73 ± 3.28 *
I + EDTA	33.29 ± 2.52	1.31 ± 1.28	34.60 ± 1.60
I + H_2_O_2_	30.37 ± 3.79 *	13.57 ± 4.33 *	43.94 ± 4.11 *

*γᵈ*: Dispersive component of surface energy; *γᵖ*: Polar component of surface energy; SFE: Surface free energy. * *p* < 0.05 versus control (I + S).

**Table 3:** Electrochemical parameters obtained from potentiodynamic polarization curves. Asterisks indicate statistically significant differences between groups (*p* < 0.05).

	Ecorr (mV)	Icorr (μA/cm^2^)	Rp (Ω/cm^2^)	CR (mm/year)
I + S	−321 ± 23	0.098 ± 0.009	1.02 × 10^6^ ± 1.10 × 10^5^	4.90 × 10^−4^ ± 7.89 × 10^−5^
I + CA	−362 ± 27 *	0.044 ± 0.005 *	1.27 × 10^6^ ± 1.33 × 10^5^ *	3.91 × 10^−4^ ± 3.44 × 10^−5^ *
I + EDTA	−318 ± 23	0.095 ± 0.005	1.00 × 10^6^ ± 1.02 × 10^5^	5.15 × 10^−4^ ± 6.66 × 10^−5^
I + H_2_O_2_	−248 ± 26 **	0.012 ± 0.023 *	0.89 × 10^6^ ± 1.10 × 10^5^ **	3.55 × 10^−4^ ± 4.01 × 10^−5^ **

Ecorr: Corrosion potential; Icorr: Corrosion current density; Rp: Polarization resistance (Ω/cm^2^); CR: Corrosion rate. A single asterisk (*) indicates a significant difference compared with I + S (*p* < 0.05), while a double asterisk (**) indicates a significant difference compared with both I + S and I + CA (*p* < 0.05).

**Table 4:** Titanium ion release (parts per billion, ppb) at different immersion times in Hank’s solution for discs subjected to various passivation treatments.

	1 Day	3 Days	7 Days	14 Days	21 Days
I + S	2.9 ± 0.4	3.7 ± 0.4	4.9 ± 1.2	6.7 ± 0.3	16.1 ± 0.5
I + CA	1.5 ± 0.2 *	2.9 ± 0.4 *	3.1 ± 0.4 *	4.8 ± 0.5 *	8.0 ± 0.5 *
I + EDTA	2.8 ± 0.8	4.5 ± 0.8	5.8 ± 0.9	10.6 ± 0.9 **	15.8 ± 1.0
I + H_2_O_2_	3.3 ± 0.9	4.9 ± 0.7	7.2 ± 0.7 **	14.7 ± 0.6 ***	23.8 ± 2.3 **

A single asterisk (*) indicates a significant difference compared with the I + S group (*p* < 0.05); double asterisks (**) indicate significant differences compared with both the I + S and I + CA groups (*p* < 0.05); triple asterisks (***) indicate significant differences compared with all other groups, including I + S, I + C, and I + EDTA (*p* < 0.05).

**Table 5:** Cell viability in the cytotoxicity assay using HFF cells, expressed as a percentage.

	1:1	1:2	1:10	1:100	1:1000
I + S	80 ± 3	82 ± 2	80 ± 3	88 ± 2	92 ± 3
I + CA	77 ± 4	82 ± 4	85 ± 5	87 ± 2	98 ± 1
I + EDTA	72 ± 2	75 ± 3	77 ± 4	80 ± 1	82 ± 2
I + H_2_O_2_	71 ± 2	77 ± 1	78 ± 4	82 ± 1	84 ± 2

**Table 6:** Cell viability in the cytotoxicity assay using SaOs-2 cells, expressed as a percentage.

	1:1	1:2	1:10	1:100	1:1000
I + S	91 ± 2	92 ± 2	94 ± 3	95 ± 2	99 ± 2
I + CA	87 ± 4	87 ± 5	89 ± 5	87 ± 6	98 ± 4
I + EDTA	83 ± 3	85 ± 3	87 ± 4	85 ± 5	90 ± 7
I + H_2_O_2_	80 ± 5	79 ± 4	88 ± 4	86 ± 4	94 ± 5

**Table 7:** Colony-forming units (CFU × 10^8^) of *P. aeruginosa* and *S. sanguinis* after 24 and 48 h under different treatment conditions.

	*P. aeruginosa*		*S. sanguinis*	
	24 h	48 h	24 h	48 h
I + S	1.19 ± 0.07	1.76 ± 0.08	1.08 ± 0.12	1.28 ± 0.20
I + CA	0.85 ± 0.09 *	0.98 ± 0.07 *	0.34 ± 0.09 *	0.46 ± 0.13 *
I + EDTA	0.81 ± 0.06 *	1.20 ± 0.05 *	0.87 ± 0.09 **	1.02 ± 0.08 **
I + H_2_O_2_	0.88 ± 0.09 *	1.10 ± 0.05 *	0.45 ± 0.13 *	0.67 ± 0.12 ***

A single asterisk (*) indicates statistically significant differences compared to the I + S (*p* < 0.05), double asterisks (**) indicate a difference compared to both the I + S and I + EDTA groups (*p* < 0.05); triple asterisks (***) indicate differences compared to all other groups under the same condition.

**Table 8:** Confocal laser microscopy percentages of *P. aeruginosa* and *S. sanguinis* proportion of dead bacteria.

	*P. aeruginosa*		*S. sanguinis*	
	24 h	48 h	24 h	48 h
I + S	30 ± 3	32 ± 5	10 ± 3	12 ± 5
I + CA	72 ± 6 *	79 ± 9 *	88 ± 7 *	75 ± 6 *
I + EDTA	35 ± 5	42 ± 7	38 ± 5 **	22 ± 9
I + H_2_O_2_	85 ± 9 *	76 ± 5 *	71 ± 10 *	69 ± 12 *

A single asterisk (*) indicates statistically significant differences compared to the I + S (*p* < 0.05), double asterisks (**) indicate a difference compared to both the I + S and I + EDTA groups (*p* < 0.05); triple asterisks.

## Data Availability

The data supporting the findings of this study are available from the corresponding author upon reasonable request.
